# Digital and offline social participation configurations and depressive symptoms and life satisfaction among older adults in China

**DOI:** 10.3389/fpubh.2026.1804015

**Published:** 2026-04-21

**Authors:** Lizhen Wei, Yu Tian, Haiquan Chen

**Affiliations:** 1 Department of Social Work, Guangdong Baiyun University, Guangzhou, China; 2 School of Business, Sun Yat-sen University, Guangzhou, China; 3 School of Management, Jinan University, Guangzhou, China

**Keywords:** depressive symptoms, digital engagement, latent class analysis, life satisfaction, mental well-being, older adults, social participation

## Abstract

**Background:**

Social participation is a key determinant of mental well-being in later life, yet population based research tends to focus on overall activity rather than on how participation is configured. In digitalized societies, unequal access to digital technologies shapes how older adults combine online and offline participation, with important but poorly understood implications for mental health.

**Methods:**

Using data from 8,427 adults aged 60 years and older in China, this study applied latent class analysis to identify patterns of digital and offline social participation based on eight indicators. Multinomial logistic regression was used to examine sociodemographic and health correlates of class membership. Multiple linear regression and covariate adjusted means were used to assess associations between participation patterns, depressive symptoms, and life satisfaction.

**Results:**

Four participation classes were identified: Digital active, Offline active, Digital–offline active, and Low participation. Participation configurations were strongly stratified by age, education, residence, and health status. After adjusting for covariates, older adults in the Digital active and Digital–offline active classes reported significantly lower depressive symptoms and higher life satisfaction than those in the Offline active class, while individuals in the Low participation class showed intermediate mental health outcomes.

**Conclusion:**

Mental well-being in later life depends not only on whether older adults are socially active but on how digital and offline participation are configured. In a digitally transforming society, digital engagement appears to function as a socially structured psychosocial resource, contributing to inequalities in mental well-being across later life.

## 1 Introduction

Population ageing is occurring alongside rapid digitization of everyday life, including communication, information access, and public and commercial services. For older adults, this shift creates a new and unequal social landscape: some are able to maintain relationships and participate in social life through both online and offline channels, while others remain restricted to offline spaces or disengage from both. As a result, patterns of social participation in later life are becoming increasingly heterogeneous. This has important public health relevance, as different patterns of digitally enabled and offline participation may be associated with different levels of mental well-being.

Social participation has long been recognized as a fundamental determinant of health in later life (1). Meta-analytic evidence indicates that stronger social relationships are associated with lower mortality risk, comparable to major biomedical and behavioral risk factors (2). Systematic review evidence further shows that social isolation and loneliness are consistently linked to poorer physical and mental health outcomes, underscoring the public health importance of social engagement (3). More recent population-based and longitudinal studies suggest that greater social participation is associated with fewer depressive symptoms and better overall mental health among older adults (4–6). These associations are particularly salient in later life, when retirement, declining health, and the loss of close social ties can reduce everyday opportunities for meaningful engagement and increase vulnerability to psychological distress.

Mental health in later life is commonly conceptualized along both negative and positive dimensions, most notably depressive symptoms and life satisfaction. Depressive symptoms capture emotional distress and psychological burden, whereas life satisfaction reflects a broader evaluation of one’s life circumstances and social integration (7, 8). Although related, these two dimensions do not necessarily move in parallel and may respond differently to social conditions. A growing body of population-based evidence shows that higher levels of social participation are associated with fewer depressive symptoms and greater life satisfaction among older adults, including studies from China and other ageing societies (9–12). These associations have been observed across multiple forms of engagement, ranging from community activities to everyday social interaction, underscoring the relevance of participation for both psychological distress and subjective wellbeing in later life (13, 14).

The meaning and structure of social participation are being reshaped by digitization. As communication, information access, and everyday services increasingly move online, digital technologies have become an integral part of how older adults maintain social ties and access social resources. Digital engagement has been shown to reduce social isolation and support psychological well-being by facilitating communication, perceived support, and functional independence, although effects vary across individuals and contexts (4, 13, 15). Rather than simply adding new activities, digital participation therefore has the potential to reorganize how older adults engage with others and with society. As a result, participation in later life may no longer be understood simply as more or less engagement. Instead, older adults may combine digital and offline activities in qualitatively different ways. At the same time, access to and use of digital technologies are socially stratified by education, income, health, and place of residence (16, 17), suggesting that digitalization may be reshaping opportunities for social participation in later life.

Despite growing interest in digital ageing, important conceptual and empirical gaps remain. Much of the existing literature examines offline social participation and digital engagement as separate domains, or relies on single indicators and summed measures of overall activity. These approaches are useful for estimating average effects, but they are less well suited to capturing the possibility that older adults combine online and offline participation in qualitatively different ways. In digitalized societies, social participation is no longer structured along a single continuum from low to high engagement. Some older adults may remain active mainly through offline channels, some may extend participation into both digital and offline domains, while others may be active online but socially limited offline or disengaged in both respects. Such combinations may carry different implications for mental well-being, yet they are difficult to identify using variable-centered approaches alone. A person-centered approach is therefore needed to examine how digital and offline participation cluster within individuals. Latent class analysis is particularly appropriate in this context because it identifies unobserved subgroups characterized by distinct configurations of participation indicators, rather than focusing only on the independent contribution of each activity. This approach allows us to move beyond the question of whether participation matters in general and instead examine how different participation profiles are patterned across the older population and whether these profiles are differentially associated with mental well-being. In this way, LCA serves not merely as a statistical technique but as an analytic strategy aligned with the study’s substantive aim. In other words, the use of LCA allows us to examine whether participation in later life is better conceptualized as a set of distinct participation configurations rather than as variation along a single continuum of overall activity.

China offers a particularly revealing context for examining these issues. Rapid population ageing is unfolding alongside one of the world’s fastest digital transformations of everyday services, communication, and social interaction, while large urban–rural and socioeconomic inequalities persist in access to digital resources (17–19). Older adults in China are increasingly expected to use smartphones and online platforms for health care, payments, transportation, and social contact, yet digital access and digital skills remain highly uneven. In this setting, digital inclusion may strongly shape opportunities for social connection, information access, and functional independence in later life, making China a critical case for understanding how digital and offline participation are jointly patterned and how these patterns relate to mental well-being.

Building on this perspective, we propose that social participation in the digital era may take the form of qualitatively distinct participation configurations that combine digital and offline activities in different ways. Accordingly, this study addresses three research questions. First, what distinct configurations of digital and offline social participation can be identified among older adults in China? Second, how are these participation configurations stratified by sociodemographic and health characteristics? Third, how are these configurations associated with depressive symptoms and life satisfaction? Based on prior research on social participation, digital inclusion, and mental well-being, we expected that older adults with higher engagement across both digital and offline domains would report fewer depressive symptoms and greater life satisfaction, whereas those with low participation across both domains would show the least favorable mental well-being profile. We also expected class membership to be socially stratified, with older age, lower education, rural residence, and poorer health associated with less digitally integrated participation patterns.

Using nationally representative data from the 2023 wave of the China Longitudinal Aging Social Survey, we therefore proceeded in three analytical steps. First, latent class analysis was used to identify configurations of digital and offline social participation based on eight participation indicators. Second, multinomial logistic regression was used to examine sociodemographic and health differences in class membership. Third, covariate-adjusted regression models and post-estimation comparisons were used to assess how the identified participation configurations were associated with depressive symptoms and life satisfaction. By linking a person-centered classification of participation to population stratification and mental well-being outcomes, this study aims to advance understanding of how participation patterns in the digital era are linked to mental well-being and social inequality in later life.

## 2 Methods

### 2.1 Data source and sample

This study used data from the 2023 wave of the China Longitudinal Aging Social Survey (CLASS), a nationally representative survey designed to examine the social, economic, and health conditions of older adults in China. CLASS collects detailed information on demographic characteristics, health status, family relations, and social participation through structured face-to-face interviews conducted by trained interviewers, during which respondents provided self-reported answers that were recorded by the interviewer. A total of 11,670 individuals were initially surveyed. After excluding respondents aged below 60 years and those with missing data on the variables used in the latent class and regression analyses, the final analytical sample consisted of 8,427 participants. Missing data on analysis variables were handled using a complete-case approach. The mean age of the sample was 70.48 years (SD = 6.24; range = 60–100), with 51.6% of respondents being male and 48.4% female. The CLASS dataset is publicly available and fully anonymized. Therefore, this secondary analysis did not require additional ethical approval.

### 2.2 Instruments

#### 2.2.1 Participation typologies

To identify distinct configurations of participation in digital and offline contexts, eight dichotomous indicators were constructed from the CLASS survey data. In contemporary digital societies, opportunities for participation increasingly depend not only on direct social interaction but also on access to digital infrastructure and the ability to use digital technologies that enable engagement with information, services, and social networks.

Accordingly, the indicators were selected to capture complementary dimensions of participation opportunities in later life. Online participation was represented using four indicators. Internet access (D17) was included to capture the availability of basic digital infrastructure that enables participation in online communication and other digital activities. Online social (D18_4_1, D18_4_2) indicated whether respondents used digital tools to communicate with friends or family. Online information (D18_4_4, D18_4_5) reflected whether respondents used the internet to obtain information relevant to daily life, such as news, health knowledge, or community announcements. Online function (D18_4_3, D18_4_8) denoted the use of digital technologies for practical purposes, including mobile payment, online shopping, and transportation applications. Although these activities involve instrumental tasks, they represent important forms of digital engagement through which older adults interact with services, institutions, and broader social systems, thereby expanding the range of participation opportunities available in contemporary digital environments.

Offline participation was measured using four indicators reflecting different forms of community involvement. Offline civic participation (D13, D13_1, D13_2) referred to general and formal civic involvement in community affairs, such as voting, expressing opinions, and public supervision. Offline governance (D14_1, D14_4) referred to active engagement in community management and problem-solving, such as safety patrols and conflict mediation. Offline helping (D14_2, D14_5) indicated informal mutual assistance, including providing care, companionship, or help to other community members. Offline leisure (D15_5, D15_6) captured participation in community-based recreational and cultural activities.

In the CLASS questionnaire, participation in these activities is measured using structured survey items. The Internet access item is originally coded as a binary response (1 = yes, 2 = no). The online activity items used to construct the three online participation indicators were originally coded as binary responses (1 = yes, 0 = no). The offline participation items are measured using ordinal frequency categories indicating how often respondents engage in the activity (0 = never, 1 = several times a year, 2 = at least once a month, 3 = at least once a week, 4 = almost every day). For the purposes of identifying participation configurations using latent class analysis, all indicators were recoded into binary variables distinguishing participation or availability from non-participation or non-availability (1 = participated or available; 0 = not participated or not available). Specifically, for the offline participation items, responses indicating any level of engagement (from “several times a year” to “almost every day”) were coded as participation, whereas “never” was coded as non-participation. This dichotomization was adopted to emphasize the conceptual distinction between participation and non-participation and to avoid sparse frequency categories among older respondents, which may reduce the stability and interpretability of latent class solutions.

#### 2.2.2 Depressive symptoms

Depressive symptoms were measured using the 9-item depressive symptoms scale from the E2 module of the CLASS questionnaire (items E2_1 to E2_9), an abbreviated CES-D measure that has been used in prior studies of Chinese older adults (20). Recent peer-reviewed research based on the 2023 CLASS data has also applied the same 9-item short CES-D measure (21). Responses were rated on a three-point Likert scale ranging from 1 (never) to 3 (often). Items E2_1, E2_4, and E2_9 were reverse-coded so that higher values consistently indicated more severe depressive symptoms. The item scores were then averaged to construct a continuous depressive symptom index. In the present sample, the scale showed acceptable internal consistency (Cronbach’s *α* = 0.775).

#### 2.2.3 Life satisfaction

Life satisfaction was assessed with a single item (B17): “Overall, how satisfied are you with your current life?” In the original CLASS questionnaire, responses were coded from 1 (very satisfied) to 5 (very dissatisfied). For ease of interpretation, the scale was reverse-coded so that higher scores indicate greater life satisfaction, ranging from 1 (very dissatisfied) to 5 (very satisfied). Previous research has shown that single-item life satisfaction measures correlate strongly with the multi-item Satisfaction with Life Scale and yield similar patterns of associations with theoretically relevant variables in large representative samples (22).

#### 2.2.4 Covariates

Covariates included sociodemographic and health characteristics known to be associated with social participation and mental health. Sociodemographic variables included age (60–74, 75–84, ≥85), gender (male, female), educational attainment (illiterate, primary or below, secondary or above), marital status (living with a spouse vs. not), and place of residence (rural, town, city). In the CLASS dataset, residence is recorded according to the administrative classification of the respondent’s community and reflects differences in settlement type commonly used in Chinese population surveys. Socioeconomic status was measured using self-rated economic status (worse, average, and better). Health-related covariates included self-rated health (poor, average, and good), presence of chronic disease (yes/no), and need for care (yes/no).

### 2.3 Data analysis

Latent class analysis (LCA) was used to identify distinct patterns of social participation among older adults based on multiple indicators of digital and offline activities. LCA models with increasing numbers of classes were estimated in Mplus version 8 using maximum likelihood with robust standard errors. Model fit was evaluated using the Akaike Information Criterion (AIC), the Bayesian Information Criterion (BIC), the sample-size adjusted BIC (aBIC), entropy, and likelihood ratio tests including the Lo–Mendell–Rubin test (LMRT) and the bootstrap likelihood ratio test (BLRT) (23). Selection of the optimal class solution was based on a combination of statistical fit, classification quality, and substantive interpretability of the resulting participation profiles (24). Posterior class membership probabilities were used to assign individuals to their most likely participation class for subsequent analyses.

To examine sociodemographic and health inequalities in participation patterns, multinomial logistic regression models were estimated in SPSS version 28, with the low participation class specified as the reference category. Odds ratios (ORs) and 95% confidence intervals (CIs) were reported to quantify the associations between individual characteristics and the likelihood of belonging to each participation class.

To assess the associations between participation patterns and mental well-being, multiple linear regression models were fitted with depressive symptoms and life satisfaction as continuous outcomes variables. Participation class was included as the key independent variable. The offline active class was used as the reference group to facilitate comparison between digitally engaged, low participation, and mixed participation profiles. All models were adjusted for sociodemographic characteristics (age, gender, education, marital status, and place of residence), subjective economic status, self-rated health, chronic disease, and need for care.

Because regression coefficients from models with categorical predictors primarily represent differences relative to a reference category, they provide limited information about the overall level of mental health outcomes across participation groups. To facilitate interpretation, covariate-adjusted means of depressive symptoms and life satisfaction were therefore estimated for each participation class using estimated marginal means (EMMeans) derived from the regression models. These adjusted means represent the expected outcome values for each participation class while holding covariates constant. Bonferroni-adjusted pairwise comparisons were then conducted to assess which participation classes differed significantly from one another.

To assess the potential impact of missing data, we conducted a sensitivity analysis using multiple imputation based on a random forest approach. Missing values in depressive symptoms, life satisfaction, and covariate variables were imputed using all available sociodemographic and participation indicators as predictors. The regression analyses were then repeated using the imputed dataset. The results were substantively consistent with those obtained from the complete-case analysis (see Supplementary Table S2).

## 3 Results

### 3.1 Sample characteristics

Table 1 summarises the characteristics of the analytic sample (*N* = 8,427). The sample was almost evenly distributed by gender (51.6% male, 48.4% female). Most participants were aged 60–74 years (78.3%), with 19.3% aged 75–84 years and 2.4% aged 85 years or older. Educational attainment was heterogeneous: 17.3% were illiterate, 41.1% had primary education or below, and 41.6% had secondary education or above. Participants were similarly distributed between rural (43.5%) and urban areas (43.2%), with 13.3% living in towns. Most respondents were living with a spouse (83.1%). In terms of socioeconomic and health characteristics, 85.2% reported an average economic status, and 58.2% rated their health as good. Chronic disease was highly prevalent (80.4%), and 6.2% reported needing daily care.

**Table 1:** Sample characteristics (*N* = 8,427).

Variables	*N* (%)
Gender	
Male	4,346 (51.6)
Female	4,081 (48.4)
Age	
60–74	6,600 (78.3)
75–84	1,628 (19.3)
≥85	199 (2.4)
Education	
Illiterate	1,457 (17.3)
Primary or below	3,466 (41.1)
Secondary or above	3,504 (41.6)
Residence	
Rural	3,668 (43.5)
Town	1,119 (13.3)
City	3,640 (43.2)
Marital status	
With spouse	7,004 (83.1)
Without spouse	1,423 (16.9)
Subjective economic status	
Better	713 (8.5)
Average	7,179 (85.2)
Worse	535 (6.3)
Self-rated health	
Healthy	4,902 (58.2)
Average	2,998 (35.6)
Poor	527 (6.3)
Care needs	
Yes	523 (6.2)
No	7,904 (93.8)
Chronic disease	
Yes	6,775 (80.4)
No	1,652 (19.6)

### 3.2 Bivariate associations among the participation indicators

Table 2 presents the pairwise phi coefficients among the eight participation indicators. Overall, the indicators were positively associated, although the strength of association varied across domains. Correlations among the online indicators were generally stronger, particularly between internet access and online social participation (*φ* = 0.757) and between internet access and online information use (*φ* = 0.736). Positive within-domain associations were also observed among the offline indicators, especially between offline civic participation and offline governance (*φ* = 0.602) and between offline civic participation and offline leisure (*φ* = 0.623). By contrast, correlations between online and offline indicators were generally weaker, for example between online social participation and offline governance (*φ* = 0.083) and between online function and offline civic participation (*φ* = 0.148). This pattern suggests that digital and offline participation were related but not reducible to a single unidimensional continuum, supporting the use of latent class analysis to identify qualitatively distinct participation configurations among older adults.

**Table 2:** Pairwise phi coefficients among the eight participation indicators.

Indicator	1	2	3	4	5	6	7	8
1. Internet access	1							
2. Online social	0.757^**^	1						
3. Online information	0.736^**^	0.713^**^	1					
4. Online function	0.483^**^	0.496^**^	0.503^**^	1				
5. Offline civil participation	0.221^**^	0.229^**^	0.198^**^	0.148^**^	1			
6. Offline governance	0.068^**^	0.083^**^	0.094^**^	0.102^**^	0.602^**^	1		
7. Offline helping	0.156^**^	0.165^**^	0.109^**^	0.043^**^	0.330^**^	0.322^**^	1	
8. Offline leisure	0.385^**^	0.384^**^	0.328^**^	0.227^**^	0.623^**^	0.175^**^	0.247^**^	1

Phi coefficients are reported because all eight participation indicators were dichotomous. All coefficients were statistically significant at *p* < 0.001.

### 3.3 Latent class analysis

Table 3 summarises the fit statistics for latent class models with one to six classes. As the number of classes increased, the AIC, BIC, and adjusted BIC (aBIC) values decreased steadily, and both the Lo–Mendell–Rubin test (LMRT) and the bootstrap likelihood ratio test (BLRT) remained significant for solutions with two to six classes, indicating incremental improvements in model fit. All candidate models showed high entropy values above 0.90, suggesting good classification quality.

**Table 3:** Model fit indices for latent class solutions.

Classes	AIC	BIC	aBIC	LMRT	BLRT	Entropy
1	85216.197	85272.511	85247.088	—	—	—
2	63759.681	63879.348	63825.325	<0.001	<0.001	0.996
3	59233.461	59416.48	59333.857	<0.001	<0.001	0.997
4	54993.322	55239.694	55128.47	<0.001	<0.001	0.965
5	54260.916	54570.64	54430.816	<0.001	<0.001	0.970
6	53735.433	54108.51	53940.086	<0.001	<0.001	0.946

Because information criteria continued to improve with additional classes, model selection was guided by the aBIC and the principle of parsimony. Although the LMRT and BLRT remained statistically significant for the 5-class and 6-class solutions, inspection of the 5-class model indicated that the additional class did not represent a substantively distinct participation pattern. Instead, it largely reflected a minor subdivision of an existing class without introducing a new interpretable configuration of participation. Therefore, considering interpretability, class size, and the diminishing improvement in aBIC beyond four classes, the four-class solution was retained. The improvement in aBIC values became progressively smaller beyond the four-class solution, suggesting diminishing gains in model fit with additional classes (Supplementary Figure S2). The four-class model also provided high classification accuracy (entropy = 0.965) and substantively interpretable and well-balanced class sizes. On this basis, the four-class solution was selected for subsequent analyses.

**Figure 1:**
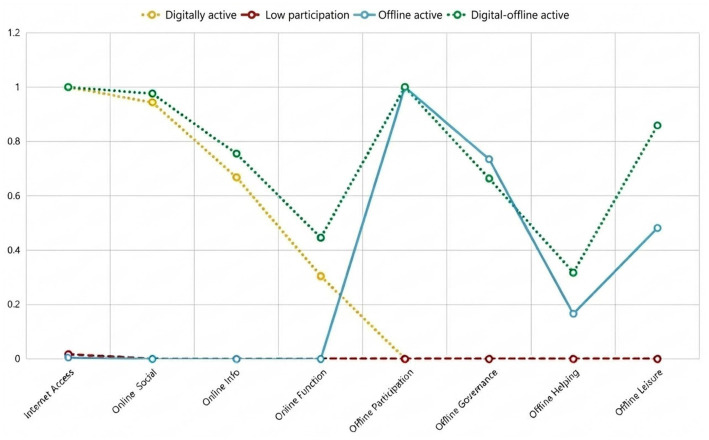
Conditional response probabilities of participation indicators across latent classes.

Figure 1 displays the conditional response probabilities for the four latent classes. Based on their distinct digital and offline participation profiles, the classes were labelled Low participation, Offline active, Digital active and Digital–offline active.

The Low participation class (17.5%, *n* = 1,482) was characterized by low engagement in both digital and offline activities.

The Offline active class (26.6%, *n* = 2,235) showed high engagement in offline participation, governance, helping, and leisure activities, but very limited digital engagement.

The Digital active class (9.3%, *n* = 773) was characterized by high engagement in internet use and online social and information activities, with little involvement in offline activities.

The Digital–offline active class was the largest group (46.6%, *n* = 3,937) and exhibited consistently high engagement across both digital and offline domains, representing a broadly active participation profile.

### 3.4 Sociodemographic and health characteristics associated with latent class membership

Table 4 reports multinomial logistic regression results for class membership, using the Low participation class as the reference group. Clear social gradients were observed across the four participation profiles.

**Table 4:** Multinomial logistic regression of the identified latent classes concerning the sociodemographic characteristics of older adults.

Variables	Offline active vs. low participation	Digital active vs. low participation	Digital–offline active vs. low participation
	OR [95% CI]	OR [95% CI]	OR [95% CI]
Gender			
Male	0.972 (0.8490–1.114)	0.947 (0.786–1.139)	0.911 (0.793–1.047)
Female (Rf.)			
Age			
60–74 years old	**1.790** **(1.292–2.480)** ^***^	**6.417** **(2.699–15.259)** ^***^	**10.293** **(5.596–18.931)** ^***^
75–84 years old	1.113 (0.803–1.541)	**2.489** **(1.040–5.955)** ^*^	**2.166** **(1.169–4.013)** ^*^
≥85 (Rf.)			
Education			
Secondary or above	**1.496** **(1.214–1.843)** ^***^	**9.888** **(6.946–14.078)** ^***^	**7.885** **(6.351–9.789** ^***^
Primary or below	1.147 (0.982–1.339)	**4.176** **(3.008–5.798)** ^***^	**2.189** **(1.819–2.635)** ^***^
Illiterate (Rf.)			
Marital status			
With spouse	1.037 (0.884–1.215)	**1.319** **(1.031–1.687)** ^*^	**2.571** **(2.126–3.109)** ^***^
Without spouse (Rf.)			
Place of residence			
City	1.012 (0.861–1.189)	**2.017** **(1.638–2.483)** ^***^	**3.028** **(2.581–3.551)** ^***^
Town	1.060 (0.870–1.292)	0.936 (0.684–1.279)	**1.919** **(2.581–3.551)** ^***^
Rural (Rf.)			
Subjective economic status			
Better	1.072 (0.776–1.481)	1.463 (0.852–2.515)	**1.814** **(1.282–2.566)** ^**^
Average	1.211 (0.963–1.524)	**2.808** **(1.827–4.315)** ^***^	**2.334** **(1.786–3.049)** ^***^
Worse(Rf.)			
Self-rated health			
Healthy	**1.355** **(1.052–1.744)** ^*^	**2.702** **(1.808–4.040)** ^***^	**3.57** **(2.691–4.738)** ^***^
Average	**1.360** **(1.063–1.740)** ^*^	**1.752** **(1.171–2.622)** ^**^	**1.925** **(1.450–2.555)** ^***^
Poor (Rf.)			
Care needs			
Yes	**0.625** **(0.472–0.827)** ^**^	**2.508** **(1.790–3.514)** ^***^	**1.481** **(1.112–1.972)** ^**^
No (Rf.)			
Chronic disease			
Yes	**1.245** **(1.037–1.496)** ^*^	0.858 (0.683–1.078)	**1.334** **(1.113–1.598)** ^**^
No (Rf.)			

Bold values indicate statistically significant results. *p < 0.05; **p < 0.01; ***p < 0.001.

Membership in the Digital–offline active class was the most strongly stratified. Compared with adults aged 85 years and older, those aged 60–74 had more than ten times higher odds of belonging to this group (OR = 10.293, 95% CI 5.596–18.931), and those aged 75–84 also had significantly higher odds (OR = 2.166, 95% CI 1.169–4.013). Educational attainment was particularly influential: older adults with secondary education or above were almost eight times more likely to be Digital–offline active than illiterate individuals (OR = 7.885, 95% CI 6.351–9.789). Urban residence further increased the likelihood of membership (OR = 3.028, 95% CI 2.581–3.551), as did reporting better (OR = 1.814, 95% CI 1.282–2.566) or average economic status (OR = 2.334, 95% CI 1.786–3.049). Good self-rated health was also a strong predictor (OR = 3.57, 95% CI 2.691–4.738), whereas needing care reduced the likelihood of belonging to this group.

A similar but more selective pattern characterized the Digital active class. Adults aged 60–74 were more than six times as likely to be Digital active as those aged 85 and above (OR = 6.417, 95% CI 2.699–15.259), and those aged 75–84 also showed elevated odds (OR = 2.489, 95% CI 1.040–5.955). Educational gradients were pronounced: secondary education or above was associated with nearly tenfold higher odds of being Digital active (OR = 9.888, 95% CI 6.946–14.078), while primary education was associated with a fourfold increase (OR = 4.176, 95% CI 3.008–5.798). City residence also substantially increased the likelihood of digital activity (OR = 2.017, 95% CI 1.638–2.483), as did better self-rated health (OR = 2.702, 95% CI 1.808–4.040). In contrast to the Digital–offline active class, economic status showed a weaker and more selective association with this profile.

The Offline active class exhibited a more limited and distinct pattern. Younger age was associated with higher odds of offline social participation (60–74 vs. ≥ 85: OR = 1.790, 95% CI 1.292–2.480), as was higher education (secondary or above: OR = 1.496, 95% CI 1.214–1.843). Good self-rated health was also a significant predictor (OR = 1.355, 95% CI 1.052–1.744). However, unlike the digitally engaged classes, offline participation was not associated with urban residence or economic status, and individuals who required care were significantly less likely to belong to this group (OR = 0.625, 95% CI 0.472–0.827).

### 3.5 Associations between participation classes and depression and life satisfaction

Table 5 presents results from multiple linear regression models examining the associations between latent participation classes and depressive symptoms and life satisfaction, adjusting for sociodemographic, economic, and health characteristics. The Offline active class was used as the reference group.

**Table 5:** Multiple linear regression analysis assessing associations between the identified latent classes and depressive symptoms and life satisfaction.

Variables	Depressive symptoms			Life satisfaction		
	B	SE	P	B	SE	P
Latent class						
Low participation	**−0.083**	**0.015**	**<0.001**	0.003	0.021	0.893
Digital active	**−0.069**	**0.019**	**<0.001**	**0.124**	**0.027**	**<0.001**
Digital–offline active	**−0.053**	**0.013**	**<0.001**	**0.132**	**0.019**	**<0.001**
Offline active (Rf.)						
Gender						
Male	**−0.031**	**0.010**	**0.001**	−0.015	0.014	0.289
Female (Rf.)						
Age						
60–74 years old	**−0.075**	**0.033**	**0.022**	**−0.127**	**0.048**	**0.008**
75–84 years old	−0.042	0.033	0.208	**−0.029**	**0.048**	**0.547**
≥85 (Rf.)						
Education						
Secondary or above	−0.027	0.016	0.081	0.030	0.023	0.186
Primary or below	0.000	0.014	0.979	−0.019	0.020	0.361
Illiterate (Rf.)						
Marital status						
With spouse	**−0.078**	**0.014**	**<0.001**	**0.075**	**0.020**	**<0.001**
Without spouse (Rf.)						
Place of residence						
City	**−0.063**	**0.011**	**<0.001**	**0.107**	**0.016**	**<0.001**
Town	−0.001	0.015	0.909	−0.003	0.022	0.903
Rural (Rf.)						
Subjective economic status						
Better	**−0.056**	**0.025**	**0.026**	**0.392**	**0.037**	**<0.001**
Average	**−0.076**	**0.02**	**<0.001**	**0.194**	**0.029**	**<0.001**
Worse (Rf.)						
Self-rated health						
Healthy	**−0.235**	**0.021**	**<0.001**	**0.790**	**0.030**	**<0.001**
Average	**−0.067**	**0.021**	**0.001**	**0.439**	**0.031**	**<0.001**
Poor (Rf.)						
Care needs						
Yes	**0.133**	**0.012**	**<0.001**	**0.060**	**0.030**	**0.046**
No (Rf.)						
Chronic disease						
Yes	**0.135**	**0.021**	**<0.001**	**−0.214**	**0.018**	**<0.001**
No (Rf.)						

Bold values indicate statistically significant results.

After adjustment, participation patterns remained significantly associated with both mental health outcomes. Notably, all three alternative participation profiles were associated with lower depressive symptoms than the Offline active class. Compared with those who were primarily engaged in offline activities, older adults in the Low participation class (*B* = −0.083, *p* < 0.001), the Digital active class (*B* = −0.069, *p* < 0.001), and the Digital–offline active class (*B* = −0.053, *p* < 0.001) reported significantly fewer depressive symptoms.

A similar but more differentiated pattern was observed for life satisfaction. Both participation profiles that involved digital engagement showed significantly higher life satisfaction than the Offline active class, including the Digital active group (*B* = 0.124, *p* < 0.001) and the Digital–offline active group (*B* = 0.132, *p* < 0.001). In contrast, the low participation class did not differ significantly from the Offline active group (*B* = 0.003, *p* = 0.893), indicating that offline-only participation was associated with life satisfaction levels comparable to social disengagement.

Among covariates, better self-rated health, higher subjective economic status, being married, and urban residence were consistently associated with lower depressive symptoms and higher life satisfaction, whereas chronic disease and care needs were associated with poorer mental health outcomes.

### 3.6 Differences in depressive symptoms and life satisfaction across participation classes

Table 6 presents covariate-adjusted means of depressive symptoms and life satisfaction across the four participation classes, holding sociodemographic, socioeconomic, and health characteristics constant. Bonferroni-adjusted pairwise comparisons were used to assess statistically significant differences between classes.

**Table 6:** Covariate-adjusted means of depressive symptoms and life satisfaction by participation class.

Latent class	Depressive symptoms			Life satisfaction		
	Adjusted mean	95% CI	Post-hoc	Adjusted mean	95% CI	Post-hoc
Digital active	1.655	[1.624–1.686]	a	3.887	[3.842, 3.932]	a
Digital–offline active	1.677	[1.662–1.692]	a	3.903	[3.881, 3.925]	a
Low participation	1.684	[1.672–1.697]	b	3.761	[3.726, 3.796]	b
Offline active	1.721	[1.702–1.740]	c	3.758	[3.730, 3.786]	b

Controlled for: age, gender, education, marital status, residence, subjective economic status, self-rated.

Health, chronic disease, and need for care. Values with different letters indicate statistically significant.

Differences between classes (Bonferroni-adjusted *p* < 0.05).

For depressive symptoms, a pronounced pattern of differentiation was observed across participation profiles. The Offline active class exhibited the highest adjusted level of depressive symptoms [M = 1.721, 95% CI (1.702, 1.740)], whereas the Digital active class showed the lowest [M = 1.655, 95% CI (1.624, 1.686)]. The Digital–offline active class had similarly low depressive symptoms [M = 1.677, 95% CI (1.662, 1.692)], while the Low participation class occupied an intermediate position [M = 1.684, 95% CI (1.672, 1.697)]. Both digitally engaged classes differed significantly from the Offline active class, whereas they did not differ from each other.

A parallel pattern was evident for life satisfaction. The Digital–offline active [M = 3.903, 95% CI (3.881, 3.925)] and Digital active classes [M = 3.887, 95% CI (3.842, 3.932)] reported the highest levels of life satisfaction. In contrast, both the Offline active [M = 3.758, 95% CI (3.730, 3.786)] and Low participation classes [M = 3.761, 95% CI (3.726, 3.796)] exhibited significantly lower levels.

Viewed together, these adjusted comparisons indicate that participation patterns involving digital engagement are associated with the most favorable mental health profiles, whereas reliance on offline-only participation corresponds to the most disadvantaged psychological position, with low participation falling in between.

## 4 Discussion

This study examined how older adults combine digital and offline forms of social participation and how these participation patterns are associated with depressive symptoms and life satisfaction. Using a latent class approach, four distinct participation configurations were identified: Digital active, Offline active, Digital–offline active, and low participation. These patterns were not only socially stratified by age, education, residence, and health, but were also systematically linked to mental well-being. Older adults whose participation involved digital engagement, either alone or in combination with offline activities, exhibited more favourable mental health profiles, characterized by lower depressive symptoms and higher life satisfaction. In contrast, those who were primarily engaged in offline social activities showed the highest levels of depressive symptoms and the lowest life satisfaction, while the low participation group occupied an intermediate position. Together, these findings indicate that in a digitally transforming society, the mental health correlates of social participation depend not only on whether older adults are socially active, but also on how their participation is configured across digital and offline domains.

The finding that older adults in the Digital active and Digital–offline active classes experienced lower depressive symptoms and higher life satisfaction is consistent with a growing body of research on digital engagement and later life well-being. Previous studies have shown that internet use and online social interaction are associated with greater emotional support, reduced loneliness, and improved psychological well-being among older adults (15, 25, 26). Our results extend this literature by showing that it is not merely digital use itself, but its integration with broader patterns of social participation, that is most strongly linked to mental health outcomes. Digital technologies provide opportunities to maintain social ties despite physical limitations, geographical distance, or shrinking offline networks, thereby helping older adults preserve a sense of connectedness and autonomy (27, 28). In addition, online access to information, services, and communication tools can strengthen perceived control and self-efficacy, which are important psychological resources in later life (29–31).

Beyond direct social interaction, digital participation may also expand older adults’ social capital and functional independence, enabling them to navigate everyday tasks, access health and public services, and remain engaged with the wider society (32, 33). Such forms of engagement are particularly relevant in contemporary contexts where many social, administrative, and commercial activities increasingly occur online. In this sense, digital participation represents not merely a supplementary channel of communication but an important pathway through which older adults may sustain both social integration and subjective well-being in a rapidly digitalizing environment.

At first glance, the finding that older adults in the Offline active class exhibited relatively higher depressive symptoms and lower life satisfaction appears to contradict a large body of research highlighting the psychological benefits of social participation in later life (4, 34). Classical perspectives such as Activity Theory suggest that maintaining high levels of social engagement contributes to successful ageing and psychological well-being (35, 36). One possible explanation relates to structural constraints shaping participation opportunities in a rapidly digitalizing society. Older adults who are active only in offline contexts may face barriers to digital participation, including limited digital literacy, financial constraints, or health-related limitations. Such constraints may also reflect underlying health or socioeconomic disadvantages that simultaneously restrict digital access and shape patterns of social participation. As a result, they may be excluded from many forms of communication, information exchange, and social interaction that increasingly take place through digital platforms. In such circumstances, offline-only participation may be accompanied by a broader sense of marginalization within a digitally transforming society. Older adults who remain disconnected from digital networks may experience feelings of being left behind or excluded from mainstream social life, which may in turn be associated with lower life satisfaction and higher depressive symptoms.

Beyond structural barriers, recent theoretical and empirical work has also emphasized that not all forms of participation are equally rewarding, and that the psychological consequences of activity depend on its social meaning, relational dynamics, and emotional demands (37–39). Participation experiences within offline settings may vary in their emotional demands and interpersonal expectations. Some forms of face-to-face social activity may involve subtle evaluative pressures or social obligations that shape how individuals experience these interactions. Rather than uniformly promoting well-being, the psychological implications of participation may therefore depend on the broader social context in which activities occur. By contrast, digitally mediated interaction can sometimes provide greater flexibility in how individuals manage communication and engagement, which may partially explain why digitally engaged older adults in this study showed more favorable mental health profiles.

Another noteworthy finding is that the low participation class did not exhibit the most adverse mental health outcomes, but instead fell between the digitally engaged and the Offline active groups. This pattern complements prior research showing that low participation in later life is often driven by health-related constraints rather than by social withdrawal or disengagement (40–42). Although reduced participation is generally associated with higher risk of depression, it may also limit exposure to socially demanding environments. Our findings therefore suggest that the relationship between participation and mental health may not be linear, but contingent on the types of social environments in which older adults are involved.

Beyond these specific mechanisms, our findings raise a broader theoretical question about how social participation should be conceptualized in a digitally transforming society. Much of the existing literature, informed by Activity Theory and related frameworks, has tended to treat social participation as a largely unidimensional construct, assuming that higher levels of activity are uniformly beneficial for psychological well-being. The present study suggests that this assumption may be incomplete in contexts where digital and offline forms of engagement coexist and interact. Our results indicate that mental health is associated not simply with how active older adults are, but with how their participation is configured across social and technological domains. In particular, the finding that offline-only participation is associated with poorer mental health than digitally mediated or mixed forms of engagement highlights a potential boundary condition of activity-based models of ageing. These results suggest that the psychological implications of participation may depend on the broader socio-technological environment in which activities occur. More broadly, our findings point to the value of moving toward a configuration-based perspective on later-life participation, which may better capture how digitization is reshaping patterns of social integration, inequality, and mental well-being in ageing societies.

## 5 Limitations and future directions

Several limitations should be acknowledged. First, this study is based on cross-sectional data, which precludes causal inference. Although participation patterns were strongly associated with depressive symptoms and life satisfaction, the direction of these relationships cannot be definitively established. Depressive states may influence how and whether older adults engage in social and digital activities. It is also possible that individuals experiencing poorer mental health may be less likely to adopt digital technologies, thereby reinforcing reliance on offline-only participation. Longitudinal and experimental studies are needed to clarify these dynamic processes. Second, social participation was measured using dichotomous indicators, which capture whether activities occurred but not their frequency, intensity, or subjective meaning. Future research should incorporate more fine-grained measures of engagement quality, duration, and motivation. Third, although the latent class approach captures heterogeneity in participation patterns, it does not account for changes over time. Future studies should examine how participation configurations evolve and how transitions between patterns affect mental health trajectories in later life. Finally, depressive symptoms were measured using the nine-item abbreviated CES-D scale embedded in the CLASS survey rather than the full standard CES-D or a clinical diagnostic instrument. Although this measure has been used in prior studies of Chinese older adults and showed acceptable internal consistency in the present sample, its abbreviated survey-based format may limit direct comparability with studies using the full CES-D or validated diagnostic instruments. Therefore, the results should be interpreted as reflecting depressive symptom severity rather than a clinical diagnosis of depression.

## 6 Conclusion

This study shows that social participation in later life is best understood not as a simple gradient of activity, but as a set of distinct configurations that combine digital and offline engagement in different ways. Using a latent class approach, we identified four participation patterns among older adults and demonstrated that these configurations are systematically associated with depressive symptoms and life satisfaction. Older adults whose participation involved digital engagement, whether alone or alongside offline activities, exhibited more favorable mental health profiles, whereas those relying primarily on offline participation showed higher depressive symptoms and lower life satisfaction. These findings challenge the long standing assumption that greater face to face activity is uniformly beneficial and underscore the importance of how participation is structured. By highlighting the central role of digital participation in shaping psychosocial resources and emotional well-being, this study contributes to a more nuanced understanding of ageing in a digitally transforming society and suggests that promoting inclusive and flexible forms of engagement may be key to supporting mental health in later life.

## Data Availability

Publicly available datasets were analyzed in this study. This data can be found here: The data used in this study were obtained from the China Longitudinal Aging Social Survey (CLASS). Data are available to researchers upon reasonable request from the CLASS data repository: http://class.ruc.edu.cn.
