# Supplementary material for: Digital and offline social participation configurations and depressive symptoms and life satisfaction among older adults in China
Source: Front Public Health. 2026 Apr 21;14:1804015. doi: 10.3389/fpubh.2026.1804015 (PMC13139004; doi:10.3389/fpubh.2026.1804015)
Supplement: Supplementary file 1 [file Table_1.docx]

Supplementary Note

To examine the robustness of the latent class solution, the latent class analysis was re-estimated using respondents with complete participation indicators (N = 10,002). The resulting class structure and item-response probabilities were substantively identical to those reported in the main analysis.

Supplementary Table S1

Robustness of latent class structure using the extended sample（N=10,002）

| **Label** | **N (%)** | **Internet Access** | **Online Social** | **Online Info** | **Online Function** | **Offline civil Participation** | **Offline Governance** | **Offline Helping** | **Offline Leisure** |
| --- | --- | --- | --- | --- | --- | --- | --- | --- | --- |
| Low participation | 1730 (17.3%) | 0.016 | 0.000 | 0.000 | 0.000 | 0.000 | 0.000 | 0.000 | 0.000 |
| Offline active | 3021 (30.2%) | 0.003 | 0.000 | 0.000 | 0.000 | 0.999 | 0.742 | 0.263 | 0.509 |
| Digital active | 840 (8.4%) | 0.999 | 0.944 | 0.65 | 0.32 | 0.000 | 0.000 | 0.000 | 0.000 |
| Digital–offline active | 4411 (44.1%) | 0.998 | 0.969 | 0.734 | 0.41 | 0.999 | 0.652 | 0.363 | 0.852 |

Supplementary Figure S1
Item−response probabilities of latent classes

Supplementary Figure S2
 Adjusted Bayesian Information Criterion (aBIC) values across latent class solutions

| Supplementary Table S2  Regression results based on multiple imputation (N = 10,002） | | | | | | |
| --- | --- | --- | --- | --- | --- | --- |
| **Variables** | **Depressive symptoms** | | | **Life satisfaction** | | |
|  | **B** | **SE** | **P** | **B** | **SE** | **P** |
| **Latent class** |  |  |  |  |  |  |
| Low participation | −0.029 | 0.010 | 0.004 | 0.140 | 0.020 | ＜0.001 |
| Digital active | −0.068 | 0.013 | ＜0.001 | 0.170 | 0.025 | ＜0.001 |
| Digital–offline active | −0.028 | 0.008 | ＜0.001 | 0.151 | 0.015 | ＜0.001 |
| Offline active (Rf.) |  |  |  |  |  |  |
| **Gender** |  |  |  |  |  |  |
| Male | −0.010 | 0.007 | 0.133 | −0.015 | 0.013 | 0.249 |
| Female (Rf.) |  |  |  |  |  |  |
| **Age** |  |  |  |  |  |  |
| 60−74 years old | −0.059 | 0.022 | 0.007 | −0.096 | 0.043 | 0.026 |
| 75−84 years old | −0.024 | 0.022 | 0.280 | 0.006 | 0.017 | 0.890 |
| ≥85 (Rf.) |  |  |  |  |  |  |
| **Education** |  |  |  |  |  |  |
| Secondary or above | −0.107 | 0.010 | ＜0.001 | 0.070 | 0.020 | 0.001 |
| Primary or below | −0.053 | 0.010 | ＜0.001 | 0.004 | 0.019 | 0.816 |
| Illiterate (Rf.) |  |  |  |  |  |  |
| **Marital status** |  |  |  |  |  |  |
| With spouse | −0.072 | 0.009 | ＜0.001 | 0.118 | 0.018 | ＜0.001 |
| Without spouse (Rf.) |  |  |  |  |  |  |
| **Place of residence** |  |  |  |  |  |  |
| City | −0.071 | 0.008 | ＜0.001 | 0.079 | 0.015 | ＜0.001 |
| Town | −0.011 | 0.011 | 0.286 | −0.016 | 0.021 | 0.442 |
| Rural (Rf.) |  |  |  |  |  |  |
| **Subjective economic status** | |  |  |  |  |  |
| Better | −0.061 | 0.016 | ＜0.001 | 0.282 | 0.030 | ＜0.001 |
| Average | −0.060 | 0.012 | ＜0.001 | 0.159 | 0.024 | ＜0.001 |
| Worse (Rf.) |  |  |  |  |  |  |
| **Self−rated health** |  |  |  |  |  |  |
| Healthy | −0.215 | 0.013 | ＜0.001 | 0.817 | 0.026 | ＜0.001 |
| Average | −0.062 | 0.013 | ＜0.001 | 0.438 | 0.026 | ＜0.001 |
| Poor (Rf.) |  |  |  |  |  |  |
| **Need care** |  |  |  |  |  |  |
| Yes | 0.080 | 0.015 | ＜0.001 | 0.063 | 0.028 | 0.024 |
| No (Rf.) |  |  |  |  |  |  |
| **Chronic disease** |  |  |  |  |  |  |
| Yes | −0.102 | 0.008 | ＜0.001 | 0.213 | 0.016 | ＜0.001 |
| No (Rf.) |  |  |  |  |  |  |

| Supplementary Table S3  Original Chinese wording, response options, and coding notes for the CLASS items used in this study | | | | |
| --- | --- | --- | --- | --- |
| **Variable Name** | **Item Code(s)** | **Original Chinese Wording** | **Original Response Options** | **Note** |
| Depression Symptoms | E2_1–E2_9 | 1.过去一周您觉得自己心情很好吗? 2.过去一周您觉得孤单吗? 3.过去一周您觉得心里很难过吗? 4.过去一周您觉得自己的日子过得很不错吗? 5.过去一周您觉得不想吃东西吗? 6.过去一周您睡眠不好吗? 7.过去一周您觉得自己不中用了吗? 8.过去一周您觉得自己没事可做吗? 9.过去一周您觉得生活中有很多乐趣(有意思的事情)吗? | 1=没有; 2=有时; 3=经常 | Items E2_1, E2_4, and E2_9 were reverse-coded before computing the mean depression |
| Life Satisfaction | B17 | 总的来说，您对您目前的生活感到满意吗？ | 1=很满意 ; 2=比较满意; 3=一般; 4=比较不满意; 5=很不满意 | Reverse-coded so that higher scores indicate greater life satisfaction. |
| Internet access | D17 | 您现在居住的房屋是否有网络信号（有线或者无线）? | 1=是；2=否 | Recoded as 1 = yes and 0 = no. |
| Online social | D18_4_1, D18_4_2 | 您一般上网做什么？ 语音、视频聊天；文字聊天 | 0=否；1=是 | Coded as 1 if either item = yes; otherwise 0 |
| Online infomation | D18_4_4, D18_4_5 | 您一般上网做什么？ 看新闻；浏览除新闻外的各类文章/信息 | 0=否；1=是 | Coded as 1 if either item = yes; otherwise 0 |
| Online function | D18_4_3, D18_4_8 | 您一般上网做什么？ 购物；交通出行 | 0=否；1=是 | Coded as 1 if either item = yes; otherwise 0 |
| Offline civic participation | D13,D13_1, D13_2 | 近三年，您是否参加过本地居民委员会/村民委员会的投票选举/意见征询/民主监督？ | 1=是；2=否 | Coded as 1 if the respondent reported participation in any of the listed civic activities; otherwise 0. |
| Offline governance | D14_1, D14_4 | 请问过去一年中，您参加以下活动的频率是? 社区治安巡逻；调解邻里纠纷 | 0=没有参加；1=一年几次；2=每月至少一次；3=每周至少一次；4=几乎每天 | Coded as 1 if either activity was reported; otherwise 0 |
| Offline helping | D14_2, D14_5 | 请问过去一年中，您参加以下活动的频率是? 照料其他老人/小孩（如帮助购物、起居照料等）；陪同聊天 | 0=没有参加；1=一年几次；2=每月至少一次；3=每周至少一次；4=几乎每天 | Coded as 1 if either activity was reported; otherwise 0 |
| Offline leisure | D15_5, D15_6 | D15.过去一年中，您参加以下活动的情况是？（不含通过网络参加的）打麻将/下棋/打牌；广场舞 | 0=没有参加；1=一年几次；2=每月至少一次；3=每周至少一次；4=几乎每天 | Coded as 1 if either activity was reported; otherwise 0 |

| Supplementary Table S4 Distribution of the eight participation indicators prior to latent class analysis | | | |
| --- | --- | --- | --- |
| **Indicator** |  | **N** | **%** |
| Internet access | No | 4711 | 47.1 |
|  | Yes | 5291 | 52.9 |
| Online social | No | 4931 | 49.3 |
|  | Yes | 5071 | 50.7 |
| Online info | No | 6211 | 62.1 |
|  | Yes | 3791 | 37.9 |
| Online function | No | 7922 | 79.2 |
|  | Yes | 2080 | 20.8 |
| Offline civil participation | No | 2561 | 25.6 |
|  | Yes | 7441 | 74.4 |
| Offline governance | No | 4881 | 48.8 |
|  | Yes | 5121 | 51.2 |
| Offline helping | No | 7602 | 76.0 |
|  | Yes | 2400 | 24.0 |
| Offline leisure | No | 4701 | 47.0 |
|  | Yes | 5301 | 53.0 |

| Supplementary Table S5 Phi coefficients among the eight participation indicators prior to latent class analysis | | | | | | | | |
| --- | --- | --- | --- | --- | --- | --- | --- | --- |
| **Indicator** | **1** | **2** | **3** | **4** | **5** | **6** | **7** | **8** |
| 1.Internet access | 1 |  |  |  |  |  |  |  |
| 2.Online social | 0.757^**^ | 1 |  |  |  |  |  |  |
| 3.Online info | 0.736^**^ | 0.713^**^ | 1 |  |  |  |  |  |
| 4.Online function | 0.483^**^ | 0.496^**^ | 0.503^**^ | 1 |  |  |  |  |
| 5.Offline civil participation | 0.221^**^ | 0.229^**^ | 0.198^**^ | 0.148^**^ | 1 |  |  |  |
| 6.Offline governance | 0.068^**^ | 0.083^**^ | 0.094^**^ | 0.102^**^ | 0.602^**^ | 1 |  |  |
| 7.Offline helping | 0.156^**^ | 0.165^**^ | 0.109^**^ | 0.043^**^ | 0.330^**^ | 0.322^**^ | 1 |  |
| 8.Offline leisure | 0.385^**^ | 0.384^**^ | 0.328^**^ | 0.227^**^ | 0.623^**^ | 0.175^**^ | 0.247^**^ | 1 |

Note. Phi coefficients are reported because all eight participation indicators were dichotomous. All coefficients were statistically significant at p < .001.

Table S6. Comparison of sociodemographic characteristics between included and excluded participants

| **Characteristic** | **Included**  **(n = 8427)** | **Excluded** | **p-value** |
| --- | --- | --- | --- |
| **Gender** |  | n=3203 |  |
| Male | 4346 (51.6%) | 1686 (52.6%） | 0.304 |
| Female | 4081 (48.4%) | 1517 (47.4%） |  |
| **Age group** |  | n=3047 |  |
| 60–74 | 6600 (78.3%) | 2428 (79.7%) | 0.288 |
| 75–84 | 1628 (19.3%) | 552 (18.1%) |  |
| ≥85 | 199 (2.40%) | 67 (2.20%) |  |
| **Education** |  | n=3011 |  |
| Illiterate | 1457 (17.3%) | 548 (18.2%) | 0.345 |
| Primary or below | 3466 (41.1%) | 1198 (39.8%) |  |
| Secondary or above | 3504 (41.6%) | 1265 (42.0%) |  |
| **R**esidence |  | n=2994 |  |
| Rural | 3668 (43.5%) | 1320 (44.1%） | 0.081 |
| Town | 1119 (13.3%) | 350 (11.7%) |  |
| City | 3640 (43.2%) | 1324 (44.2%) |  |

Note. Values are presented as n (%) based on non-missing observations for each characteristic. Accordingly, denominators may vary across variables because of item-level missingness, particularly among excluded participants. P-values are based on Pearson chi-square tests using available cases.
